# Is Infantile Hemangioma a Neuroendocrine Tumor?

**DOI:** 10.3390/ijms23095140

**Published:** 2022-05-05

**Authors:** Priscilla Kaulanjan-Checkmodine, Sandra Oucherif, Sorilla Prey, Etienne Gontier, Sabrina Lacomme, Maya Loot, Marijana Miljkovic-Licina, Muriel Cario, Christine Léauté-Labrèze, Alain Taieb, François Moisan, Hamid Reza Rezvani

**Affiliations:** 1 BRIC, UMR 1312, Inserm, University Bordeaux, F-33076 Bordeaux, France; priscilla.kaulanjan@gmail.com (P.K.-C.); ouch_sandra@hotmail.fr (S.O.); sorilla.prey@chu-bordeaux.fr (S.P.); muriel.cario-andre@u-bordeaux.fr (M.C.); christine.labreze@chu-bordeaux.fr (C.L.-L.); alain.taieb@u-bordeaux.fr (A.T.); 2 Service de Dermatologie Adulte et Pédiatrique, CHU de Bordeaux, F-33000 Bordeaux, France; 3 Electron Microscopy Unit, Bordeaux Imaging Center, F-33076 Bordeaux, France; etienne.gontier@u-bordeaux.fr (E.G.); sabrina.lacomme@u-bordeaux.fr (S.L.); 4 CHU de Bordeaux, Service de Chirurgie Pédiatrique, F-33000 Bordeaux, France; maya.loot@chu-bordeaux.fr; 5 Department of Pathology and Immunology, University of Geneva Medical School, Rue Michel-Servet 1, CH-1211 Geneva, Switzerland; marijana.licina@unige.ch; 6 Centre de Référence pour les Maladies Rares de la Peau, CHU de Bordeaux, INSERM U1312, F-33000 Bordeaux, France

**Keywords:** hemangioma, propranolol, betablocker, catecholamines, adrenergic, telocytes

## Abstract

Infantile hemangioma (IH) is the most common infantile tumor, affecting 5–10% of newborns. Propranolol, a nonselective β-adrenergic receptor (ADRB) antagonist, is currently the first-line treatment for severe IH; however, both its mechanism of action and its main cellular target remain poorly understood. Since betablockers can antagonize the effect of natural ADRB agonists, we postulated that the catecholamine produced in situ in IH may have a role in the propranolol response. By quantifying catecholamines in the IH tissues, we found a higher amount of noradrenaline (NA) in untreated proliferative IHs than in involuted IHs or propranolol-treated IHs. We further found that the first three enzymes of the catecholamine biosynthesis pathway are expressed by IH cells and that their levels are reduced in propranolol-treated tumors. To study the role of NA in the pathophysiology of IH and its response to propranolol, we performed an in vitro angiogenesis assay in which IH-derived endothelial cells, pericytes and/or telocytes were incorporated. The results showed that the total tube formation is sensitive to propranolol only when exogenous NA is added in the three-cell model. We conclude that the IH’s sensitivity to propranolol depends on crosstalk between the endothelial cells, pericytes and telocytes in the context of a high local amount of local NA.

## 1. Introduction

Infantile hemangioma (IH), a benign vascular tumor, is the most common tumor in infants [1] affecting 5 to 10% of newborns [2]. It is characterized by a proliferative phase with a rapid abnormal growth of blood vessels, followed by a slow spontaneous involutive phase. Nevertheless, 15% of cases require intervention, depending on the IH’s location, in the event of a vital threat or functional risk [3]. Following our serendipitous discovery [4] and confirmative clinical trial [5,6], the non-selective beta-adrenergic receptor (ADRB) antagonist propranolol is currently the first-line therapy for severe/complicated IHs. Other infantile capillary tumors, such as rapidly involuting congenital hemangiomas (RICH), non-involuting congenital hemangiomas (NICH) and pyogenic granulomas, do not respond to propranolol [3,7]. Although several mechanisms, such as vasoconstriction, endothelial cell apoptosis, and/or altered angiogenesis [8,9], have been proposed to account for the benefit of propranolol in IHs, the precise mechanism of action of propranolol is still poorly understood. Identification of this mechanism may improve therapies based on betablockers and the development of more targeted drugs, not only for IHs, but also for cancer.

Our aim was to determine whether the therapeutic action of propranolol counteracts the pathological activation of the beta-adrenergic pathway. We postulated that competition between the propranolol and catecholamines, the natural agonists of ADRB, might be the principal mechanism of action of propranolol. Of note, catecholamines are known to play a role in the pathophysiology of neurological, psychiatric, metabolic and cardiovascular disorders, and more recently they were shown to affect angiogenesis, notably during carcinogenesis [10]. For instance, the activation of ADRB by noradrenaline (NA) in macrophages resulted in the abundant production of proangiogenic factors, such as MMP-2, MMP-9 and VEGF [11,12]. NA-mediated activation of ADRB leads to the synthesis of interleukin-6 in ovarian tumor cell lines [10]. The binding of NA to ADRB can affect tumor angiogenesis by triggering cAMP, PKA or NOTCH pathways [11,13,14]. Propranolol has an inhibitory effect on these signaling pathways through direct binding to the ADRBs [8,13,15]. Pan et al. showed that propranolol inhibits the NA-induced increase in cyclins A2 and D2, thereby affecting the cell cycle progression and proliferation [11]. Lastly, it has been shown that an ADRB2 knockout impairs angiogenesis in mice and that an ADRB2 blockade reduces the expression of proangiogenic VEGF [16]. Altogether, it makes sense to speculate that competition between the propranolol and catecholamines in IHs can affect cell proliferation and angiogenesis.

Since there are no optimal animal models, nor any IH cell lines, to study the mechanism of action of propranolol, we isolatedIH-derived endothelial cells, pericytes and telocytes (IH-EC, IH-PER and IH-TC, respectively) to develop an in vitro IH model responsive to propranolol. Although IH-EC express ADRB1-2 [17] similar to normal dermal endothelial cells [17,18,19,20,21,22], whether they can synthesize catecholamines as their normal counterparts do remains elusive [16]. IH-PER, which surround EC in proliferating IHs [23,24], have been also shown to express ADRB1-2 [17,25]. TC, which are unique stromal cells present in normal dermis (TC skin) in the close vicinity of vascular structures, affect angiogenesis through auto- and paracrine signaling via their long, thin, typical prolongations named telopodes [26]. Moreover, we recently showed that IH-TC, which express ADRB2, play a critical role in the pathophysiology of IHs [27].

## 2. Results

### 2.1. Propranolol Treatment Reduces NA Level in IH

**Figure 1:**
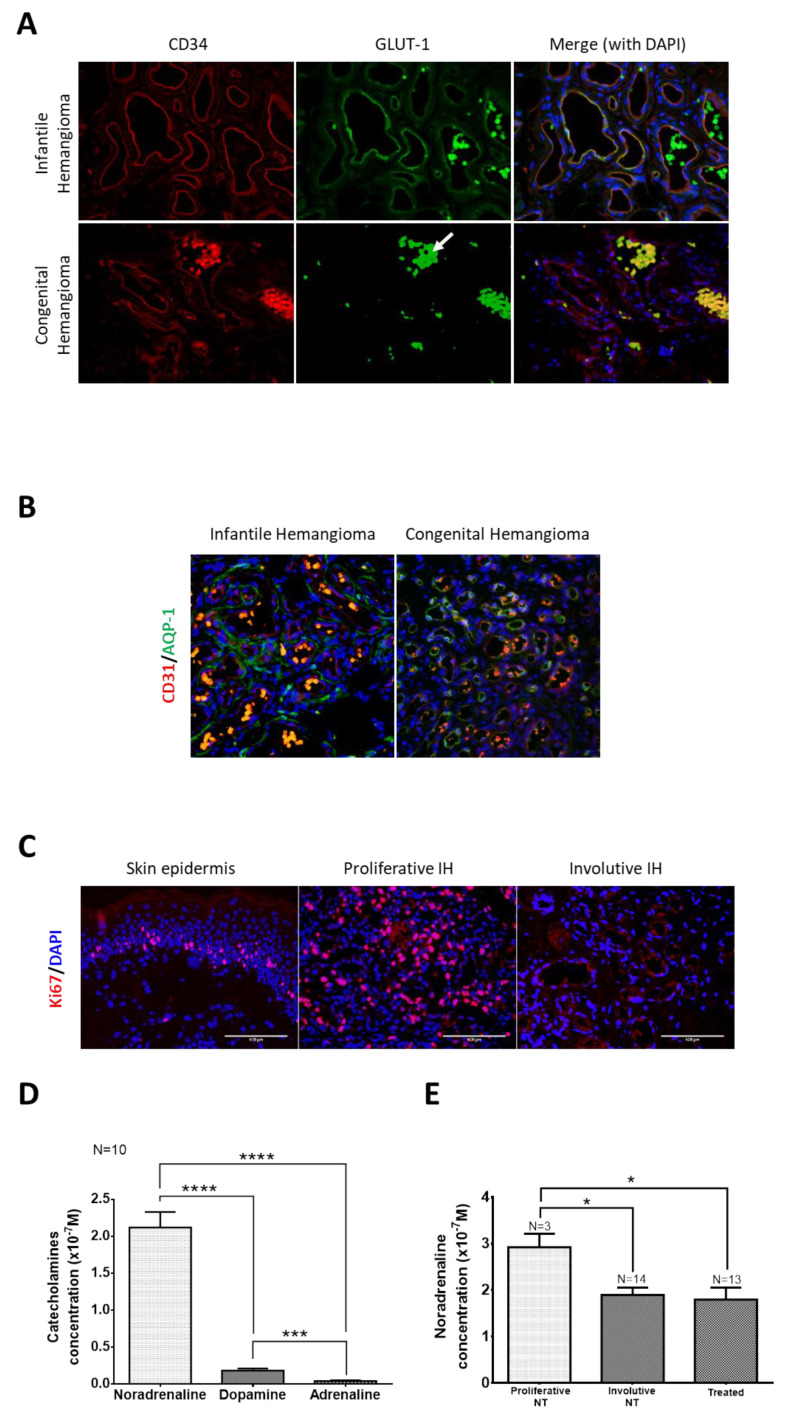
Propranolol treatment reduces noradrenaline level in proliferative IHs. (**A**) Characterization of IHs compared to congenital hemangiomas by GLUT-1 immunofluorescence staining. Red blood cell (white arrows) staining is used as positive control. Endothelial cells in IHs express GLUT-1 but not in congenital hemangiomas. (**B**) Characterization of IH with AQP1 staining. Telocytes in IH tissue express AQP1 compared to congenital hemangiomas where it is expressed in EC. (**C**) Characterization of proliferative and involutive IHs by Ki67 immunofluorescence staining. Skin epidermis (left panel) is used as positive control. Numbers of Ki67+ cells are significantly higher in the proliferative IH than in the involutive IH. Scale bar 100 µm. (**D**) Levels of NA, dopamine and adrenaline measured in IH tissues by ELISA. (**E**) NA levels in the untreated (NT) proliferative or involutive IH as well as in the propranolol-treated (Treated) IH were measured by ELISA. Data are presented as mean ± SEM. * *p* < 0.05, *** *p* < 0.01, **** *p* < 0.0001. NA: noradrenaline, IH: infantile hemangioma.

Because propranolol is an antagonist of ADRB, we wondered whether the levels of the natural agonists of ADRB, namely NA, adrenaline and dopamine, are upregulated in IHs. To test this hypothesis, we first measured the levels of catecholamines in propranolol-treated and untreated IH tissues at the proliferative and involutive stages (Figure 1). Of note, the clinical diagnosis of IH tissues used for this study was confirmed by GLUT-1 staining, which is widely used as a diagnostic marker [28], and by AQP1 staining, which has been recently proposed as a marker of antitumor propranolol response [27]. In contrast to congenital hemangiomas, IHs expressed GLUT-1 and AQP1 in their EC and TC, respectively (Figure 1A,B). Furthermore, Ki67 immunostaining was performed to distinguish between proliferative and involutive IH tissues. The number of Ki67+ cells was much higher in the proliferative IH than in the involutive IH (Figure 1C). Considering the NA, adrenaline and dopamine IH concentrations, only significant amounts of NA were found in the IH tissues (Figure 1D). Dopamine was detectable at low levels, and adrenaline was observed at a level close to the detection threshold (Figure 1D).

The NA levels decreased in spontaneously involuted IHs compared to untreated proliferative IHs. Interestingly, propranolol treatment significantly decreased the NA concentrations when taken during the tumor proliferative phase, while it had no significant effect on the NA level when administrated during the involutive phase (Figure 1E).

Altogether, our results indicate that propranolol decreases the concentration of NA in IHs when started at the proliferative phase and that spontaneous involution of IH is accompanied by a decrease in the NA tissue concentration.

### 2.2. Catecholamine Biosynthetic Enzymes Are Expressed by IH Cells and Their Expression Levels Are Reduced by Propranolol

**Figure 2:**
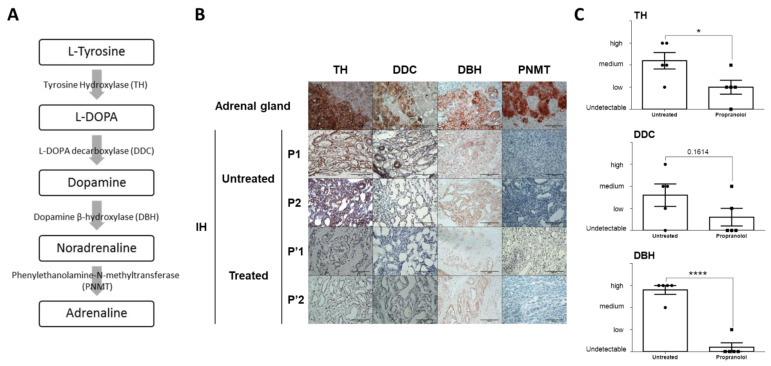
Enzymes implicated in catecholamine biosynthesis pathway are expressed in IH tissues and their expressions are decreased by propranolol treatment. (**A**) Catecholamine biosynthesis pathway is shown. (**B**) All catecholamine biosynthesis enzymes except PNMT are expressed in IH tissue, enabling them to synthesize dopamine and NA. Expression levels of these enzymes are reduced with propranolol treatment. The adrenal gland is used as a positive control. Expression levels of catecholamine biosynthesis enzymes in untreated patients are different from treated patients. Two representative pictures of 5 untreated patients and 5 propranolol-treated patients are shown. Scale bar size is 100 µm. NA: noradrenaline, IH: infantile hemangioma. (**C**) Immunostained sections were scored considering the intensity of TH, DDC and DBH in a blinded manner for 5 untreated patients and 5 propranolol-treated patients. Data are represented as mean ± SEM, * *p* < 0.05, **** *p* < 0.0001.

Given the presence of catecholamines in IH tissues, we wondered whether the enzymes implicated in the catecholamine biosynthesis pathway were expressed by IH cells. As illustrated in Figure 2A, tyrosine hydroxylase (TH), the rate-limiting enzyme of catecholamine synthesis, catalyzes the hydroxylation of tyrosine to L-DOPA. The latter is converted to dopamine by L-DOPA decarboxylase (DDC). Dopamine is hydroxylated by dopamine-β-hydroxylase (DBH) to NA, which is finally methylated to adrenaline by phenylethanolamine N-methyltransferase (PNMT). Immunohistochemistry analyses showed that TH, DDC and DBH, but not PNMT, were expressed by IHs (Figure 2B). Furthermore, the expression levels of TH, DDC and DBH in propranolol-treated IHs were much lower than those found in untreated proliferative IHs (Figure 2C).

**Figure 3:**
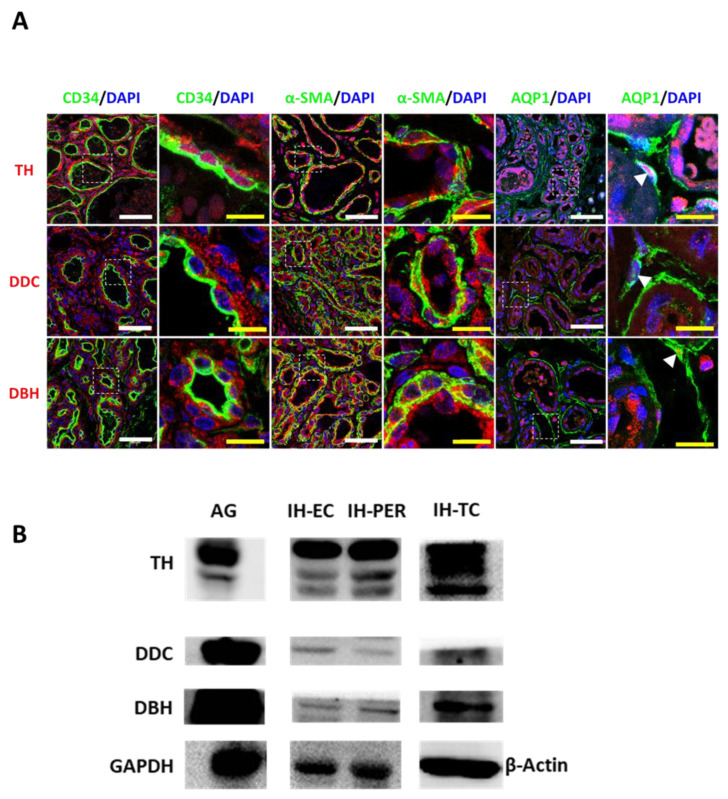
Catecholamine biosynthesis enzymes are expressed in IH-EC, -PER and -TC. (**A**) Immunofluorescence staining of TH, DDC and DBH in IHs. Endothelial cells are characterized by CD34-positive staining, pericytes by α-SMA and telocytes by AQP1 antibodies. Nucleus is stained with DAPI. The white scale bar size is 50 µm, and the yellow scale bar is 10 µm. (**B**) TH, DDC and DBH expression levels in the different cell types isolated from IHs were evaluated by Western blot. The adrenal gland (AG) is used as a positive control. GAPDH or β-actin are used as loading controls. IH: infantile hemangioma.

We then wondered which cells express these enzymes in IHs. Since immunohistochemistry of catecholamine enzymes showed perivascular staining (Figure 2B), EC, PER and TC were respectively immunostained with anti-CD34, anti-αSMA and anti-AQP1 antibodies and then co-immunostained with the antibodies against catecholamine biosynthetic enzymes. Results showed that all three cell types (EC, PER and TC) expressed TH, DDC and DBH (Figure 3A). In addition, these cells were sequentially sorted from IH tissues using anti-CD31, anti-CD34 and anti-CD146 magnetic microbeads (Supplemental Figure S1A), as described in the Material and Methods section. The isolated cells were then characterized with immunostaining (Supplemental Figure S1B,C). Placental pericytes and HUVEC were used as the control. A Western blot analysis of the expression of the catecholamine biosynthetic enzymes confirmed the expression of TH, DDC and DBH by all these cells (Figure 3B and Figure S2), suggesting that they are able to synthesize catecholamines in vitro.

Altogether, our data indicate that IH-EC, -PER and -TC express the first three enzymes of the catecholamine biosynthetic pathway and that the levels of these enzymes in propranolol-treated IHs were lower than the levels found in untreated IHs.

### 2.3. IH Sensitivity to Propranolol Depends on Crosstalk between IH-EC, PER and TC and the Presence of a Threshold Level of NA

**Figure 4:**
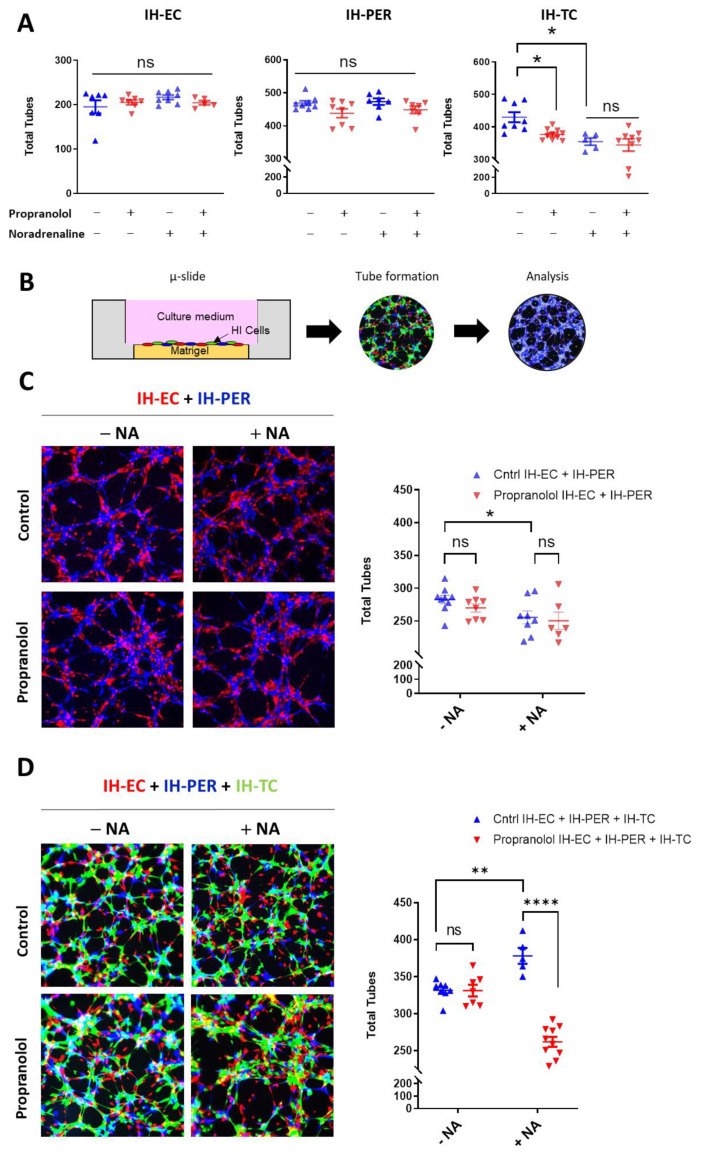
A threshold level of NA and a crosstalk between EC, PER and TC are the prerequisite for detecting the antiangiogenic effect of propranolol on our in vitro Matrigel tube formation assay. (**A**) IH-EC, IH-PER and IH-TC were seeded separately into Matrigel-coated wells. The formations of tube-like structures were imaged 3 h later and quantified using Wimasis image analysis system. (**B**) Schematic showing the in vitro tube formation assay using two or three different cell types. Each cell type is labeled with a distinct fluorescent probe. Here, EC were labeled in red, PER in blue and TC in green. Tube formations were then surveyed and imaged. The images were finally analyzed with Wimasis image analysis system. (**C**) Left panel is a representative image illustrating tube formation by IH-EC and IH-PER 3 h after their seeding into Matrigel-coated wells containing propranolol and/or NA. Quantification of total formed tubes is shown in right panel. (**D**) Left panel is a representative image illustrating tube formation by IH-EC, IH-PER and IH-TC (1:1:1 ratio) at 3 h after their seeding into wells. Quantification of total tubes (right panel) showed that NA promotes total tube numbers and this promotion is abolished if propranolol is added. Data are represented as mean ± SEM, ns: non-significant, * *p* < 0.05, ** *p* < 0.01, **** *p* < 0.0001. IH: infantile hemangioma, EC: endothelial cells, PER: pericytes, TC: telocytes, NA: noradrenaline.

To study the role of NA in the pathophysiology of IHs and the response to propranolol, we used capillary-like tube formation assays on Matrigel matrices in which IH-EC, -PER and/or -TC were incorporated. When used separately, IH-EC, PER and TC were all able to form tubes in vitro (Figure 4A and Figure S3). However, their capacity to form tubes was not affected upon treatment with propranolol, even when exogenous NA was added (Figure 4A). Of note, propranolol or NA treatment has a very moderate effect on the total tubes formed by IH-TC (Figure 4A). An IH-EC and PER co-culture model was then used to investigate whether there is a difference in the capacity of the capillary-like tube formation between the cells isolated from an IH when treated with propranolol in the presence or absence of exogenous NA (Figure 4B,C). The results showed that the total tubes formed in the presence of both IH-EC and -PER were not sensitive to propranolol in the presence or absence of exogenous NA (Figure 4C). Finally, we used a capillary-like tube formation assay in which the three cell types (IH-EC, PER and -TC) were incorporated to create a 3D model closer to the pathophysiology of IHs. Results showed that the total tubes formed in the three-cell model increased in the presence of exogenous NA, and the propranolol treatment resulted in a significant reduction in the total tubes in the latter condition (Figure 4D).

Altogether, our results indicate that the combination of three IH-derived cell types (i.e., EC, PER and TC) and exogenous NA is critical for detecting an efficient inhibitory effect of propranolol on the capillary-like tube formation in vitro.

### 2.4. Proliferative IH Have the Competency of Neuroendocrine Secretion

**Figure 5:**
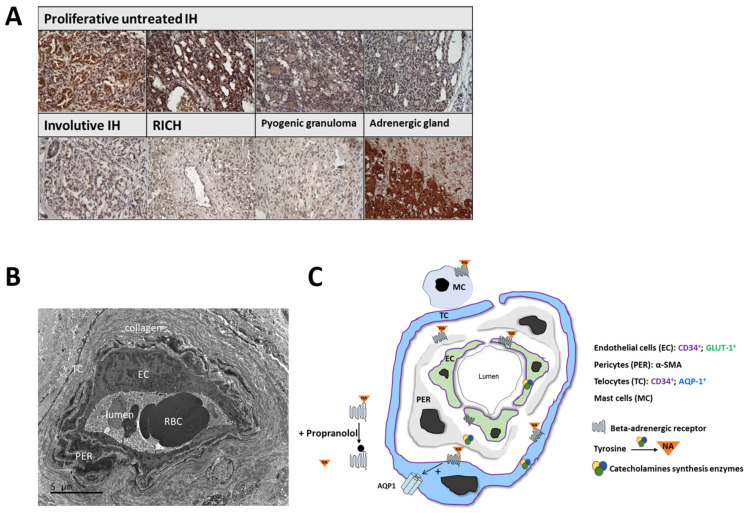
Proliferative IH tissues present some features of neuroendocrine tumors. (**A**) Immunostaining for Chromogranin A (CgA) counterstained with hemalun. CgA-positive cells appear in brown. Scale bar size is 100µm. While high expression levels of CgA are detected in untreated proliferative IHs, low levels are detected in involutive IHs and other vascular tumors, including rapidly involutive congenital hemangiomas (RICH) and pyogenic granulomas. The adrenal gland used as a positive control. (**B**,**C**) Model outlining IH responses to propranolol. (**B**) Electron microscopy of IH vessel showing endothelial cells surrounded by pericytes and telopodes of telocytes in perivascular space. Scale bar size is 5 µm. (**C**) IH-EC, -PER and -TC express the first three enzymes involved in catecholamine biosynthesis pathway, enabling them to release NA locally. Our previous data [17,27] demonstrated that beta-adrenergic receptors are present on IH-EC, -PER, -TC and mast cells. It is likely that NA-mediated activation of ADRB in an autocrine and/or paracrine manner results in activation of proangiogenic signaling pathways in different cell types, leading to dramatic growth and development of blood vessels. Propranolol antagonizes a proangiogenic NA effect through binding to ADRB receptors. NA: noradrenaline, IH: infantile hemangioma.

Given the expression of catecholamine biosynthesis enzymes by IH tissues and the critical role of NA levels in creating an in vitro angiogenesis model with sensitivity to propranolol treatment, we wondered whether IHs possess adequate transporters for the paracrine secretion of catecholamine, as neural tissues do. To answer this question, we examined the expression of chromogranin A (CgA) in IH tissues. Of note, as a member of the granin family of proteins, CgA is one of the major components of the secretory granules of most endocrine and neuroendocrine cells. This protein, whose immunohistochemical detection represents one of the most widely used markers of neuroendocrine cells, is expressed by normal and tumor cells of the diffuse endocrine and neuroendocrine systems, as well as in some cancer cells that can undergo neuroendocrine differentiation. CgA is involved in granulogenesis and the processing of secretory protein cargo prior to secretion [29,30,31,32]. Immunostaining analyses of CgA expression in IH tissue revealed high expression levels of CgA in proliferative untreated IH. Of interest, CgA was detectable at very low levels in involutive IHs and congenital hemangiomas (Figure 5A).

Altogether, these results suggest that proliferative IH tissues present some of the features of neuroendocrine tumors.

## 3. Discussion

A specific feature of IH tissues is the presence of high concentrations of NA, which are efficiently lowered by propranolol treatment. Given that there is no established in vitro model for studying the pathophysiology of IHs and the mechanism underpinning the efficacy of propranolol, we developed a simplified IH-like model with IH-PER, IH-EC and IH-TC isolated from the same biopsy. To detect an antiangiogenic effect of propranolol in this model, a high local concentration of NA is a prerequisite.

Due to the presence of high levels of NA in the proliferative IH tissue and the expression of TH, DDC and DBH in IH-PER, IH-EC and IH-TC, our results strongly suggest the local production of NA in IH tissue. In agreement, it has been shown that normal EC can synthesize NA [16]. The expression of catecholamine synthesis enzymes was clearly decreased in IH tissues treated with propranolol. Propranolol is known to prevent NA fixation on adrenergic receptors; however, it is not clear how the NA levels decrease during IH regression. Two possible explanations are as follows: First, the decrease in NA may be due to the upregulation of catecholamine degradation enzymes, such as catechol-O-methyl-transferase (COMT) or monoamine oxidase (MAO). In accordance with this mechanism, Jacob et al. showed that propranolol treatment in rats induces a significant increase in COMT and MAO [33]. Second, propranolol treatment may reduce the expression or the activity of the catecholamine synthesizing enzymes. In agreement with this hypothesis, Tuross and Patrick reported that propranolol has a direct inhibitory effect on the activity of TH, the rate-limiting step in the biosynthesis pathways of catecholamines in the striatal and hypothalamic synaptosomes [34]. Srivastava and Kapoor also reported that propranolol reduces DBH activity and, consequently, NA levels in the rat brain [35].

We have previously shown that beta-adrenergic receptors in IH tissues are expressed in EC, TC and PER with a concomitant higher expression in mast cells [17,27]. In the current study, we found that NA, which can act as a proangiogenic factor in the regulation of angiogenesis in IH tissue, can be produced locally by IH-derived EC, TC and PER. The need to add exogenous NA to create an appropriate model responding to propranolol suggests that other cells, such as mast cells, as well as any circulating factors not present in vitro, could intervene in the regulation of the system. The NA locally produced in IHs could activate an autocrine/paracrine angiogenic loop via ADRB present on the surface of various IH cell types (Figure 5). In agreement with our results, growing evidence indicates that the activation of the ADRB in several types of cancers can promote tumor angiogenesis by triggering the production of proangiogenic factors. For instance, Thaker et al. showed that ADRB2 activation in ovarian carcinoma cell lines via the extrinsic stress-induced release of catecholamines results in the upregulation of VEGF, which in turn promotes tumor vascularization and aggressive growth [36]. Other angiogenic factors such as MMP-2, MMP-9 and IL-6 can also be elevated via the ADRB signaling pathway following stimulation by adrenaline or NA in several breast cancer cell lines [37]. In such tumor models, ADRB antagonists, including propranolol, have been shown to reduce the expression of these proangiogenic factors [36,37,38]. Furthermore, the specific knockdown of signal transduction at the ADRB2 level in vascular ECs can directly impair angiogenesis in vivo and tubulogenesis in vitro [18,39,40]. Our data suggest that intrinsically produced NA in IHs can drive tumor development. Consistent with this hypothesis, it has been shown that the expression of several proangiogenic factors, such as VEGF, MMP2, MMP9 [41] and HIF-1α [42], which are highly upregulated in IHs during the proliferative phase, decrease after tumor involution. Better understanding of the therapeutic propranolol mechanism related to hemangioma pathophysiology and time course may improve clinical management and multidisciplinary approaches. Indeed, there are still unmet needs especially when several complications occur such as ulceration, relapses after treatment stop and/or prolonged course.

In conclusion, we demonstrate that the presence of NA in pharmacological amounts in IH tissues is dramatically limited by propranolol during the growth phase of IHs. We therefore suggest that the high local production of NA is a specific feature of IHs and the major driver of its development.

## 4. Materials and Methods

### 4.1. Infantile Hemangioma and Foreskin Tissues

This study was performed on 54 consecutive patients undergoing surgery for an IH (none of them had PHACE syndrome), as well as one congenital hemangioma (RICH from a 3-day-old male patient) and one pyogenic granuloma (an 11-year-old male patient) at Bordeaux Children’s Hospital between 2015 and 2021, in accordance with the Declaration of Helsinki (Supplementary Table S1). All samples were obtained after consent from the patients or their family/legal guardians. The Ethics Committee of Bordeaux University Hospital approved the study (CER-BDX 2021-47). The clinical diagnosis of IH was confirmed by immunohistochemical detection of GLUT-1.

### 4.2. Catecholamines ELISA Assay

Catecholamine levels were analyzed by an ELISA kit (3-CAT Research ELISA, ImmuSmol, Bordeaux, France) according to the manufacturer’s instructions. Adrenaline, NA and dopamine levels were measured on 30 IH tissues. Ten mg of tissue were digested in a solution containing 100 µL of EDTA (1 mM) and sodium metabisulfite (4 mM) and then sonicated. After centrifugation at 12,000 rpm for 15 min at 4 °C, the supernatant was used for the ELISA.

### 4.3. Immunohistochemistry

Immunohistochemical stains were performed on formalin-fixed and paraffin-embedded 3-µm sections. Re-hydration and antigen retrieval were done by heating the slides in a pH 6 EDTA buffer (PT Module Buffer^®^, Thermo Scientific, Waltham, MA, USA) in a PT-link^®^ instrument (Dako, Agilent, Santa Clara, CA, USA) for 20 min at 97 °C. All the primary antibodies were incubated overnight at 4 °C, and Alexa fluor 488- or 555-conjugated secondary antibodies were incubated for 1 h at room temperature. Cell nuclei were visualized by 4′, 6′-diamidino-2-phenylindole (DAPI) staining.

Samples were incubated with primary antibodies against GLUT-1, Ki67, CD34, α-SMA, AQP1 and catecholamine synthesis enzymes (TH, DDC, DBH and PNMT). References, manufacturers and concentrations of primary antibodies are listed in the Supplementary Table S2. All images were captured with the NIS-Elements software (Nikon, V3.22.10) coupled with an Eclipse epifluorescent microscope (Nikon) at a magnification ×40.

### 4.4. Protein Extraction and Western Blotting

One mg of IH tissue (*n* = 30) was digested in 100 µL RIPA lysis buffer containing 25 mM Tris-HCl pH 7.6, 150 mM NaCl, 1% NP-40, 1% sodium deoxycholate (SDS), 1 mM phenylmethanesulfonylfluoride fluoride (PMSF) and protease inhibitor cocktail (Sigma-Aldrich, St. Louis, MO, USA). After sonication, homogenates were centrifuged at 12,000 rpm for 15 min at 4 °C. The concentration of extracted protein was measured with the Pierce BCA protein assay kit (Thermo Fisher, Waltham, MA, USA). Equal amounts of total proteins were boiled in Laemmli buffer for 5 min at 90 °C, resolved by 10% sodium dodecyl sulfate polyacrylamide gel electrophoresis (SDS-PGE) and then electrophoretically transferred to polyvinylidene difluoride (PVDF, Dominique Dutscher, Bernolsheim, France) membranes. After blocking with 5% nonfat dry milk 0.5% Tween 20, membranes were incubated with primary TH, DDC, DBH, PNMT, GAPDH and β-actin antibodies. References, manufacturers and concentrations of primary antibodies are listed in the Supplementary Table S2. Then, the membranes were incubated with an appropriate horseradish peroxidase-conjugated secondary antibody (Vector) at room temperature for 1 h. Blots were finally developed using chemiluminescence ECL reagent (Clarity™ Western ECL Substrate, Biorad, Hercules, CA, USA). Images were obtained using a Fujifilm camera (Las3000, Fujifilm, Tokyo, Japan). Quantification was performed with ImageLab (Biorad). Data are presented as the relative protein level after normalization with β-actin or GAPDH.

### 4.5. IH Cell Sorting and Culture

After mechanical disruption of fresh IH, congenital hemangioma and foreskin tissues, single cell suspensions were obtained by using an enzyme cocktail composed of collagenase 1× (Sigma-Aldrich) and dispase 1× (Sigma-Aldrich) in sterile PBS. Cells of interest (IH-EC, IH-TC and IH-PER) were then isolated by sequential sorting using magnetic-activated cell sorting (MACS) based on magnetic beads coupled with antibodies (Miltenyi, Tokyo, Japan). IH-EC were first isolated with anti-CD31, then IH-TC with anti-CD34 and, finally, IH-PER with anti-CD146 antibodies (Miltenyi). Cells were seeded at a density of 100,000 cells/cm^2^ in fibronectin (Promocell, Heidelberg, Germany)-coated culture plates. EC were cultured in endothelial growth medium (EGM2, Promocell), TC in DMEM (Gibco, Grand Island, NY, USA) supplemented with 10% fetal bovine serum and PER in pericyte growth medium (Promocell), all of which was supplemented with 1% penicillin-streptomycin.

### 4.6. Immunocytofluorescence

Cells were seeded on a fibronectin-coated slide in a 48-well plate. After methanol-ethanol (*v*/*v*) fixation, cellular membranes were permeabilized with the permeabilization reagent 0.2% Triton X-100 in PBS. Nonspecific binding sites were blocked with 10% FBS. Cells were then incubated with primary antibodies against CD31, CD146, α-SMA, NG2 and PDGFRβ. Cell nuclei were counterstained with DAPI. References, manufacturers and concentrations of primary antibodies are listed in the Supplementary Table S2. All image acquisitions were done with the NIS-Elements software (Nikon, Tokyo, Japan, V3.22.10) coupled with a Leica epifluorescent microscope.

### 4.7. Electron Microscopy

Electron microscopy studies were conducted at the Bordeaux Imaging Center, Bordeaux University, a core facility of the France Bio Imaging network. IH biopsies were fixed with 2.5% (*v*/*v*) glutaraldehyde in 0.1 M phosphate buffer (pH 7.4) for 1 h at room temperature (RT). Samples were washed in 0.1 M phosphate buffer and post-fixed in 1% (*v*/*v*) osmium tetroxide in phosphate buffer 0.1 M for 2 h, in the dark at RT. Then, the tissues were dehydrated by a series of graded ethanol washes and embedded in a mixture of pure ethanol and epoxy resin (Epon 812; Delta Microscopy, Toulouse, France) 50/50 (*v*/*v*) for 2 h and then in 100% resin overnight at RT. The resin was polymerized over a period between 24–48 h at 60 °C. Samples were then sectioned using a diamond knife (Diatome, Biel-Bienne, Switzerland) on an ultramicrotome (EM UC7, Leica Microsystems, Vienna, Austria). Ultrathin sections (65 nm) were picked up on copper grids and then stained with uranyl acetate and lead citrate. Grids were examined with a transmission electron microscope (H7650, Hitachi, Tokyo, Japan) at 80 kV.

### 4.8. In Vitro Matrigel Tube Formation Assay

Matrigel-based angiogenesis assay was performed according to a previously described protocol [27]. Briefly, Matrigel^®^ Growth Factor Reduced (GFR) basement membrane matrix (Corning, NY, USA) was thawed at 4 °C. Ten μL of Matrigel were added to each well of a µ-slide angiogenesis (Ibidi), and allowed to polymerize for 30 min at room temperature and then for 1 h at 37 °C. IH-EC, IH-PER and IH-TC were labeled with CMRA548 red dye, CellTrace405 violet dye and CMFDA488 green dye (ThermoFisher), respectively. IH-derived cells were suspended in 50 µL of EGM2 containing 10 µM of NA (Mylan, Saint-Priest, France) and/or 3 µM of propranolol (Karnodyl, Primius Lab Ltd., London, UK). Different cell types were seeded separately or together in the same proportions onto Matrigel in triplicate (10,000 cells/well or 3200 IH-EC + 3200 IH-PER + 3200 IH-TC/well). The µ-slides were incubated for 3 h at 37°. After incubation, the formation of tube-like vessels was imaged at 10× magnification using an Eclipse epifluorescent microscope (Nikon). Angiogenesis parameters, including total tubes, total tube length, percentage of covered area, total loops and nets were analyzed using the Wimasis image analysis system (Onimagin Technologies, Córdoba, Spain). The mean value of three images per well was obtained for each condition in triplicate.

### 4.9. Statistical Analyses

Comparisons between two groups were calculated with GraphPad Prism V6 software (GraphPad Inc., San Diego, CA, USA) using the unpaired Student’s t-test (two tailed) or the Mann–Whitney test when the variance was not homogeneous. A *p*-value < 0.05 (*) was considered significant. Results are presented as means ± standard error of mean (SEM).

## Data Availability

The authors declare that the data supporting the findings of this study are available within the paper and its Supplementary Information files.

## Supplementary Materials

The supplementary materials can be downloaded at: https://www.mdpi.com/article/10.3390/ijms23095140/s1.
