# Supplementary material for: Is Infantile Hemangioma a Neuroendocrine Tumor?
Source: Int J Mol Sci. 2022 May 5;23(9):5140. doi: 10.3390/ijms23095140 (PMC9104933; doi:10.3390/ijms23095140)
Supplement: Supplementary file 1 [file ijms-23-05140-s001.zip › ijms-1604571-supplementary.pdf]

Supplementary Information for

Is Infantile Hemangioma a Neuroendocrine Tumor?

Priscilla Kaulanjan-Checkmodine [1,†], Sandra Oucherif [1,†], Sorilla Prey [1,2], Etienne Gontier [3], Sabrina Lacomme [3], Maya Loot [4], Marijana Miljkovic-Licina [5], Muriel Cario [1,6], Christine Léauté-Labrèze [1,2,6], Alain Taieb [1,2,6], François Moisan [1,*,†] and Hamid Reza Rezvani [1,6,*,†]

1 BRIC, UMR 1312, Inserm, University Bordeaux, F-33076 Bordeaux, France; priscilla.kaulanjan@gmail.com (P.K.-C.); ouch_sandra@hotmail.fr (S.O.); sorilla.prey@chu-bordeaux.fr (S.P.); muriel.cario-andre@u-bordeaux.fr (M.C.); christine.labreze@chu-bordeaux.fr (C.L.-L.); alain.taieb@u-bordeaux.fr (A.T.)
2 Service de Dermatologie Adulte et Pédiatrique, CHU de Bordeaux, F-33000 Bordeaux, France
3 Electron Microscopy Unit, Bordeaux Imaging Center, F-33076 Bordeaux, France; etienne.gontier@u-bordeaux.fr (E.G.); sabrina.lacomme@u-bordeaux.fr (S.L.)
4 CHU de Bordeaux, Service de Chirurgie Pédiatrique, F-33000 Bordeaux, France; maya.loot@chu-bordeaux.fr
5 Department of Pathology and Immunology, University of Geneva Medical School, Rue Michel-Servet 1, CH-1211 Geneva, Switzerland; marijana.licina@unige.ch
6 Centre de Référence pour les Maladies Rares de la Peau, CHU de Bordeaux, INSERM U1312, F-33000 Bordeaux, France

* Correspondence: francois.moisan@u-bordeaux.fr (F.M.); hamid-reza.rezvani@u-bordeaux.fr (H.R.R.)

† These authors contributed equally to this work.

*** Corresponding Authors and lead contacts**
Postal address: INSERM U1035, Bordeaux, F-33000 France
Email address: françois.moisan@u-bordeaux.fr, hamid-reza.rezvani@u-bordeaux.fr
Phone: +33-557-575-683 Fax: +33-557-571-374

Supplemental figures

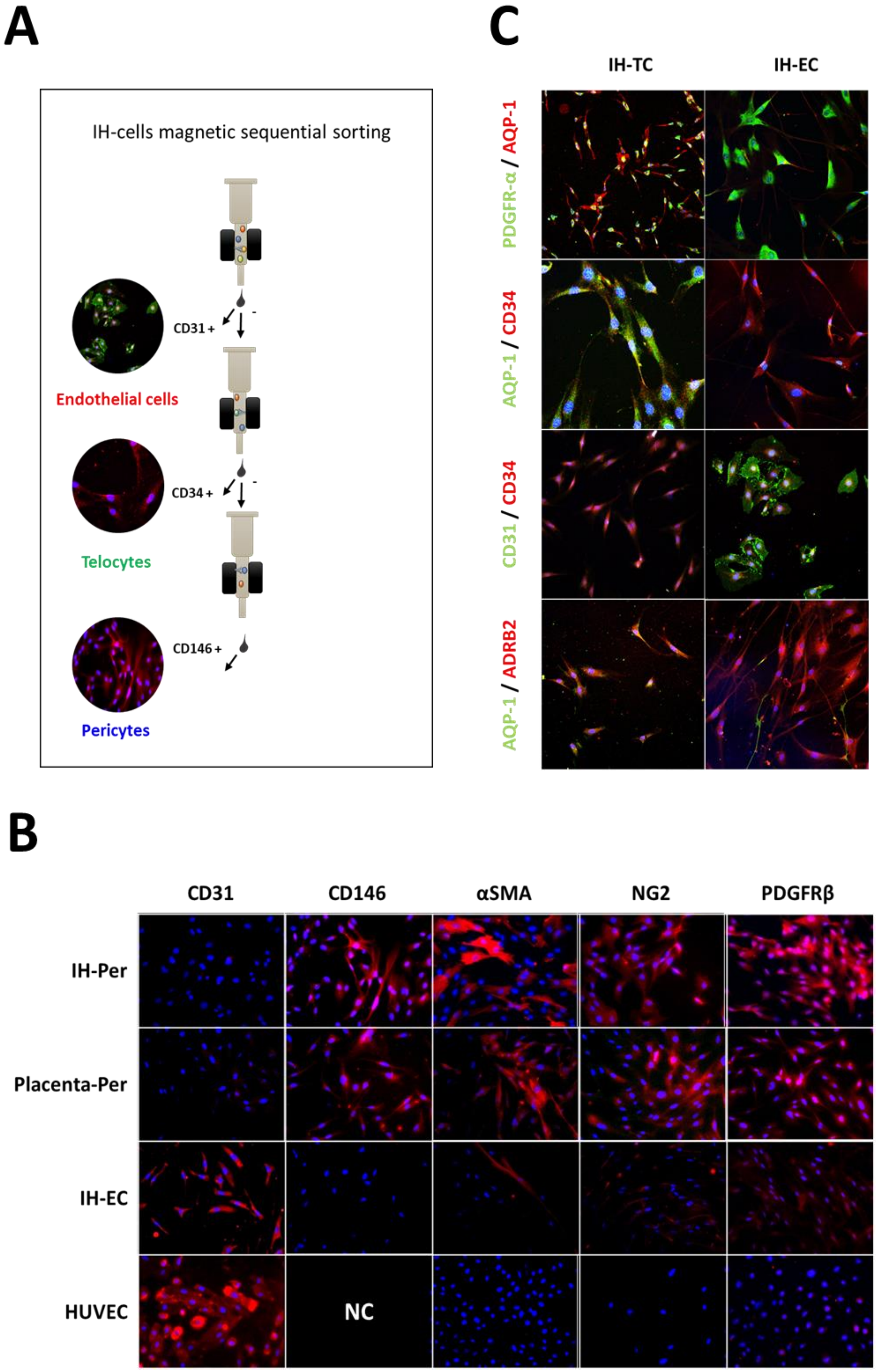

Figure S1. Isolation and characterization of EC, PER and TC from IH fresh tissue. (A) EC, TC and PER were sequentially isolated from digested IH tissue using respectively anti-CD31, anti-CD34 and anti-CD146 magnetic microbeads. **(B)** Immunocytofluorescent staining were used to verify the purity of isolated cells. IH-PER highly express CD146, αSMA, NG2 and PDGFRβ. IH-EC highly express CD34 marker. Placenta pericytes and human umbilical vein endothelial cells (HUVEC) are control cells. Nuclei are in blue with DAPI. Scale bar size is 50 µm. **(C)** Immunocytofluorescent staining were used for characterization of telocytes isolated from fresh IH tissue. IH-TC are CD31- PDGFR-CD34+ AQP1+ cells while EC are CD31+ PDGFR+CD34+ AQP1- cells.

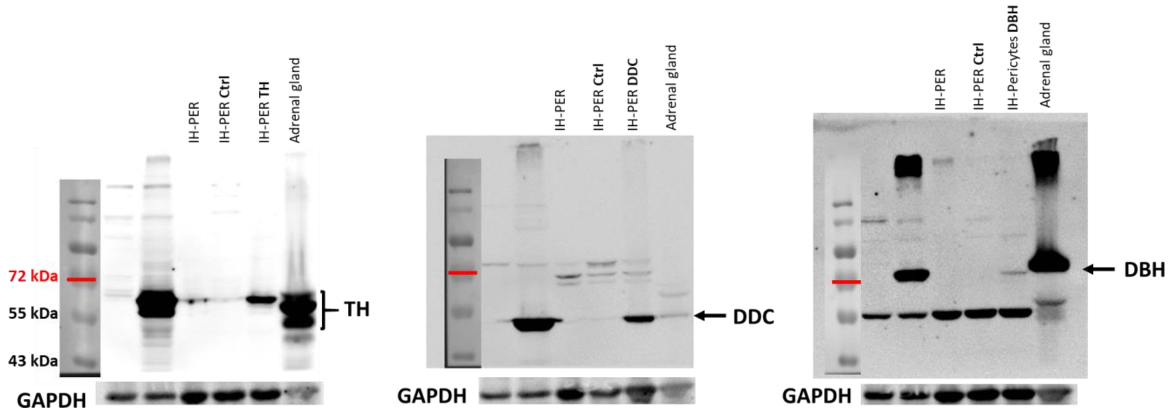

Figure S2: Western-blotting the first three enzymes of catecholamines biosynthesis pathway TH, DDC and DBH. To correctly verifying our antibodies, IH-PER were transfected with expression plasmids encoding human TH (58kDA), DDC (53 kDa) or DBH (70kDa). Protein extracts from transfected and non-transfected cells were subjected to western blotting. Adrenal gland (AG) is used as positive control. GAPDH is used as loading control. Used exposure times do not always permit identification of the endogenous protein.

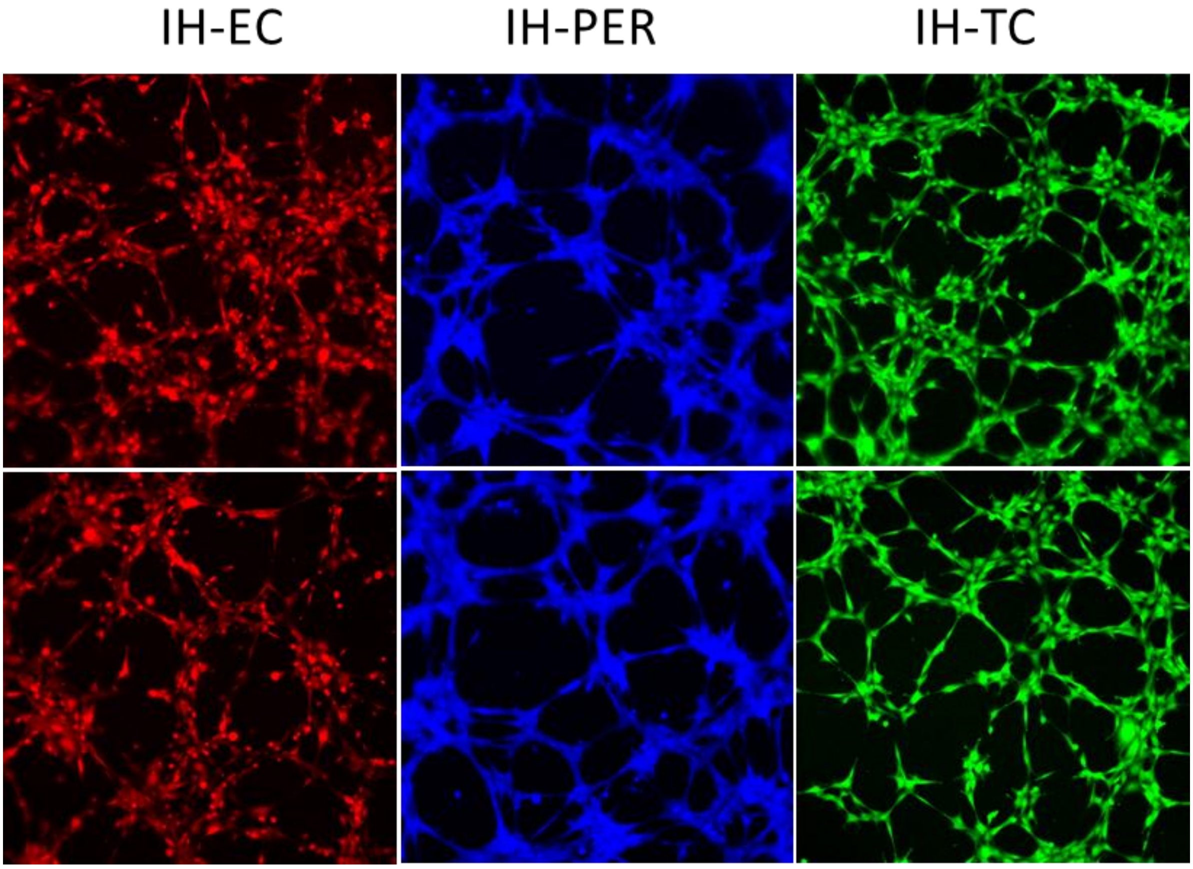

Figure S3: IH-EC, -PER and –TC are capable to form capillary-like tube when seeded into Matrigel-coated wells. IH-EC (red), IH-PER (blue) and IH-TC (green) isolated from fresh IH form tubes 3 hours after seeding in Matrigel-coated wells containing EGM2 medium.

Supplementary Tables

Table S1. List of patients included in the study

Number	Age (months)	Gender	Localization	Treatment
1	4	F	Neck	-
2	4	F	Ass	-
3	6	M	Retro-auricular	-
4	7	M	Left flank	-
5	7	F	Back	-
6	8	F	Torso	-
7	11	F	Axillary	-
8	12	M	Tempe	-
9	13	F	Pelvis	-
10	13	M	Postauricular	-
11	19	F	Temple and chin	-
12	21	F	Back	-
13	23	M	Cheek	-
14	25	F	Lips	-
15	27	M	Abdomen and elbow	-
16	28	M	Forehead	-
17	32	F	Arm	-
18	32	F	NA	-
19	33	F	Shoulder	-
20	36	F	Forehead	-
21	38	F	Cheek	-
22	41	M	Left neck	-
23	43	M	Scalp	-
24	45	F	Back	-
25	55	F	Hand	-
26	57	F	Thorax	-
27	60	F	Right flank	-
28	69	F	Right upper lip	-
29	69	M	Back	-
30	72	F	Forehead	-
31	75	F	Thorax	-
32	83	M	Torso	-
33	94	M	Torso	-
34	110	F	Thorax	-
35	123	F	Neck	-
36	131	F	Torso	-
37	4	F	Cervical anterior	Propranolol
38	6	F	Scapular	Propranolol
39	7	M	Iliac crest	Propranolol
40	9	F	Lumbar	Propranolol
41	12	M	Hemangiomatosis	Propranolol
42	18	M	Left arm	Propranolol
43	23	M	Upper lips	Propranolol
44	25	F	Shoulder	Propranolol
45	26	F	Interbrow	Propranolol
46	27	F	Forehead	Propranolol
47	30	F	Forearm	Propranolol
48	33	F	Periorbital Temporal	Propranolol
49	33	F	Axillary	Propranolol
50	33	F	Eyebrow	Propranolol
51	43	M	Forehead	Propranolol
52	59	F	Eyebrow	Propranolol
53	80	M	Lips	Propranolol
54	110	F	Back	Propranolol

Table S2. Primary antibodies used for immunohistochemistry and western blot

Antibody	Reference	Provider	Concentration	Incubation	Experimentation
Glut-1	PA1-21041	Thermofisher	1/200	Overnight 4°C	HIC
Ki67	NCL-L-Ki67-MM1	Leica	1/200	Overnight 4°C	HIC
DAPI	D9542	Sigma-Aldrich	1/10 000	15 minutes RT	HIC
TH	SAB4502964	Sigma-Aldrich	1/200*-1/1 000**	Overnight 4°C	HIC*, WB**
DDC	HPA017742	Sigma-Aldrich	1/200*-1/500*	Overnight 4°C	HIC*, WB**
DBH	PA5-34664	Thermofisher	1/200*-1/5 000*	Overnight 4°C	HIC*, WB**
PNMT	ab119784	Abcam	1/50*-1/500**	Overnight 4°C	HIC*, WB**
CD34	MA1-10202	Thermofisher	1/200	Overnight 4°C	HIC
α-SMA	ab7817	Abcam	1/500	Overnight 4°C	HIC
β-actin	A2228	Sigma-Aldrich	1/10 000	Overnight 4°C	WB
GAPDH	#2118S	Cell signaling	1/10 000	Overnight 4°C	WB
CD31	M0823	Dako	1/20	Overnight 4°C	HCC
CD146	#2505-1	Epitomics	1/250	Overnight 4°C	HCC
NG2	MAB2585	R&D systems	1/20	Overnight 4°C	HCC
PDGFRβ	sc-339	Santa Cruz	1/50	Overnight 4°C	HCC
AQP1	sc-25287	Santa cruz	1/50	Overnight 4°C	HCC
AQP1	AB2219	Merk	1/1000	Overnight 4°C	HIC
Chromogranine A	M0869	Dako	1/100	Overnight 4°C	HIC

RT: Room Temperature, Concentration: * = HIC, ** = WB
